# Transformational vs. Transactional Deployment of Intelligence

**DOI:** 10.3390/jintelligence9010015

**Published:** 2021-03-16

**Authors:** Robert J. Sternberg

**Affiliations:** Department of Human Development, Cornell University, Ithaca, NY 14853, USA; rjs487@cornell.edu

**Keywords:** intelligence, IQ, giftedness, transactional giftedness, transformational giftedness

## Abstract

The late James Flynn, to whom this Special Issue is dedicated, suggested that what will matter most to the future of the world is not levels of intelligence but rather how intelligence is deployed. In this article, I argue that we can distinguish between transactional and transformational deployments of intelligence. Loosely following Flynn, I suggest that we need to pay much more attention to the latter rather than the former.

## 1. Introduction

The late James Flynn, to whom this symposium is dedicated, is best known for discovering the so-called “Flynn effect,” by which IQs rose worldwide roughly two standard deviations during the 20th century (Flynn 1984, 1987). However, Flynn had many insights, at least one of which is arguably just as important as his insight regarding the Flynn effect. This insight was that the progress of civilization in the 21st century would depend more on how intelligence is deployed than on how much of it any of us has (Flynn 2007, 2013). In its measurement of IQ as well as scores on a variety of standardized tests used for various admissions, financial aid, employment, and other purposes, society focuses on the “amount” of intelligence. However, the future of the world depends, as Flynn recognized, not on those with the highest intelligence but rather on those who use their intelligence to the best ends.

## 2. Transactional versus Transformational Giftedness

In some of my recent work, I have distinguished between transactional and transformational giftedness (Sternberg 2020a, 2020b, n.d.) These concepts are borrowed from the literature on kinds of leadership, in which leadership theorists have distinguished between transactional and transformational leaders (e.g., Bass 1998; Bass and Avolio 1994).

I have defined *transformational giftedness* as giftedness that is transformative—transformationally gifted individuals seek positively to change the world in some way at some level. They try to make the world a better place. Thus, transformational giftedness focuses on positive and meaningful change, following a model proposed in previous work, ACCEL—active and concerned citizenship and ethical leadership (Sternberg 2017, 2019a). This model was proposed as a way to make education at all levels more responsive to the world’s needs.

*Transactional giftedness* is giftedness that is based on exchange of resources. It is tit-for-tat in nature, with an individual seeking personal benefit in exchange for some amount of effort devoted toward a societally sanctioned endeavor. Society makes its contribution by identifying particular young people as gifted or talented or at least as having high potential. Those young people then are given augmented resources, such as admission to higher tracks in public schools, or to special prestigious public schools, private schools, colleges, universities, and later, first jobs. In return, those identified as “gifted” or “talented” are expected to graduate on time, earn good grades, earn honors, succeed in the prestigious jobs they enter, earn good incomes, and the like. Transactionally gifted people thus are successful based on their personal accomplishments, not on what they have achieved for others Those transactionally gifted individuals who do not render these accomplishments are viewed as having failed to live up to their potential. They are the unfortunate detritus of an otherwise successful system of funneling people into appropriate channels for their abilities and talents. To the extent that they fulfill their transactional expectation, they help create a correlation between predictions of their success and their attained success.

Transactionally and transformationally gifted individuals have different motivational systems. Transformationally gifted individuals deploy their intelligence and other germane abilities into making a positive, meaningful, and transformative difference; transactionally gifted individuals deploy their intelligence and other abilities to achieve gain for themselves and those who are important to them. They focus on societal and professional systems of reward and punishment. The transactionally gifted individual may make a positive difference; but they do so because of the personal rewards that may accrue because of their thoughts and actions. The transformationally gifted are not averse to such rewards, but what drives them is the positive, meaningful, and transformative difference they make.

Transactional and transformational giftedness are not mutually exclusive categories within a person: Gifted individuals are not one or the other. Rather, these kinds of giftedness are partly situational. Transformationally gifted individuals are transactional when they need to be but are transformational when the opportunity arises or when they can create the opportunity. Transactionally gifted individuals rarely or never perceive or create the opportunities in which they can be transformational.

Although I have focused on giftedness, the concepts of transactional and transformational giftedness apply at any level of intellectual ability. Basically, a transaction is an exchange between people. *Intelligence*, *as we currently measure it, is transactional*. An individual answers questions on a test. That individual is given credit each time they produce an answer that the test-creator deems to be correct. If the individual is credited with enough correct answers, the individual is described as highly intelligent. If the individual does not answer so many questions correctly, the individual is deemed to be not so intelligent. Either way, the currency of the transaction is correct answers from the examinee to questions posed by the examiner, and the rewards that ensue from the correct answers.

James Flynn’s insight was that what matters most, at least, going forward, is not the amount of the transaction, but what is done with it—how the resources are deployed. Our societies have created educational and occupational systems based on an illusion—that just having more of something will somehow generate better outcomes. However, resources do not always predict societally useful deployment of those resources. For example, there are many wealthy people, with lots of money, who use the money only for their own benefit, and who actually leverage that money to seek benefits for themselves at the expense of the less fortunate. Similarly, the world is full of people with elevated IQs who leverage their IQs for their own benefit but who make little effort to do good for the world. They simply do not prioritize whether they do or do not. Or they may make things in the world worse.

## 3. What Does Transactional Intelligence Buy Society?

Ever since the early 20th century, there have been countless psychologists who have hoped that somehow having an IQ or other score based on a standardized test of academic abilities somehow would translate into meaningful contributions to society. This kind of wishful thinking started with Terman (1916, 1925) and continues to the present day (Sternberg et al. n.d.), with the promoters of this legacy themselves beneficiaries of a system that funneled them through, in part because of their high scores on standardized tests. The legacy with which we have been left is not so different from that which, at various points in history, gave enormous preferences and benefits to people who were white, male, high-caste, or whatever, and then pointed out that they tended to be more successful; the system failed to take into account that the preferred groups were given the additional resources to succeed that others who did not have the preferred attributes were not given. In fact, the correlation between status variables and later success was not “discovered,” but rather, in large part, “created” by societal systems of reward and punishment that separated those with more of the desired attributes from those with less of them. It is perhaps too easy to sing the praises of a system that has allowed one to attain the status to sing those praises.

IQ and level of education are highly correlated (Ceci 1996). In particular, there appears to be an increase of about 1.9 points of IQ per year of education (Tommasi et al. 2015). However, Senior (2020) pointed out just how little meaning IQ, and the superior education it can bring, have for one society, that of the U.S. More than one third of current members of the U.S. House of Representatives have a law degree; more than half of Senators do. Twenty-one House members and four House members have MD degrees, and the exact same numbers have some other kind of doctorate in each chamber. In all, 95% of House members and 100% of Senators have at least a bachelor’s degree. How have these highly educated politicians performed?

The 116th Congress (current as of when I am writing) enacted 252 laws. That is by a substantial margin the fewest laws of any Congress since 1973 (when my source—https://www.govtrack.us/congress/bills/statistics--first started compiling records). This is not a political statement—it is simply a fact representing a record weak level of accomplishment for the most educated Congress ever (Burgat and Hunt 2018).

As Sandel (2020) has pointed out, the problem is that advances in education and, I would add, the corresponding increases in IQ, do not seem to buy good governance. What instead this alleged meritocracy buys us is a largely hereditary aristocracy of the highly educated, much as we have had with people who are of a particular race, sex, caste, or religion, many of whom have argued for the merit of the particular group to which they happen to belong. The problem is not limited to politicians. Some of the privileged become politicians, others become whatever, including psychologists studying whatever, including intelligence.

A point similar to Senior’s was made by Halberstram (1993) in pointing out how some of the best minds in the U.S. got us into the quagmire of the Vietnam War. Higher education, IQ, school achievement—they had all of that and more, but they did not deploy their intelligence in an effective and transformational way. Rather, they acted foolishly (Sternberg 2005) because intelligence provides no protection whatsoever against foolishness.

Carnes and Lupu (2015), in a study of 228 countries between 1875 and 2004, extended these findings. They found that college education of leaders did not mean reductions in inequality, increased G.D.P., fewer strikes, reduced unemployment, or fewer military conflicts. In other words, education seems to have bought leaders of governance little or nothing.

Perhaps those aspects of intelligence as measured by IQ fare better in predicting success in domains other than leadership. However, rather than asking this question, we might ask the question James Flynn posed, which is essentially one of whether we should not pay more attention to how intelligence is deployed rather than to how much intelligence, measured by IQ or anything else, people have.

## 4. Operationalizing Transformational Giftedness

My colleagues Aakash Chowkase, Ophélie Desmet, Sareh Karami, Jenna Landy, Jennifer Long, Jialin Lu, and I are currently engaged in research using a first-pass 2020 version of a transformational-giftedness scale (Sternberg Transformational Giftedness Scale) (see Table 1). This measure is presented not as a validated scale but rather as an indication of an operationalized direction we believe the study of the transformational deployment of intelligence can take. We are construct-validating the scale against measures we expect to converge (e.g., adaptive intelligence—Sternberg 2019b, 2021) and ones we expect to diverge (e.g., conventional tests of intelligence).

Our goal is not to measure intelligence. It is rather to measure how young people hope to deploy their intelligence in the future. We are only now starting to collect data, but I publish the scale here in its preliminary form in the hope others might seek to explore use of the scale or its analogs. Our goal is to show that transformational deployment of intelligence cannot only be conceptualized but also can be operationalized. Ultimately, we would like to link the scale to transformational accomplishments in society. Again, I emphasize that this research is only just beginning.

## 5. Conclusions

Ever since Terman’s time, many (but certainly not all) psychologists, especially in the gifted field, have been “apologists” for testing of IQ and related constructs as largely sufficient in themselves for identifying the intellectually gifted. This enterprise has helped to create an enormous self-fulfilling prophecy, whereby those who are identified as being gifted or talented or precocious, or whatever, are given the resources to succeed. Those who are not identified are not given comparable resources. A funnel is created that benefits those who already are genetically and environmentally benefited, for example, by well-educated parents. The environmental funnel augments the effects of what may be small genetic differences (Dickens and Flynn 2001), or even earlier environmental ones. The beneficiaries then write about the virtues of the system, drawing on data from correlations that reflect the system their society has in part created, and encourage generations of the future to continue to do the same. They illustrate well the fundamental principle of interpersonal attraction that we tend to value and find attractive those who are most like ourselves (see, e.g., Bradbury and Karney 2019). Each generation believes that whatever criteria they use—race, sex, religion, caste, IQ, or whatever—provides an excellent basis for establishing a true meritocracy (Gould 1981; Sternberg 1997)—and provides empirical evidence from a rigged system of socioeconomic opportunities and stratification to prove their point. Meanwhile, others and their progeny are left behind to continue the cycle across generations.

In previous work (e.g., Sternberg 1997, 2019b), I have suggested that IQ is insufficient as a measure of intelligence and that we also need to measure creative, practical, and wisdom-based skills (Sternberg 2019b, 2021). That is *not* my point in this article. Rather, my point is that whatever is used as a measure of intelligence, we should follow Flynn (2013) in emphasizing instead of levels of intelligence, rather, deployment of intelligence. In the end, what matters is what you do with your intelligence. With all the problems we have today, what the world needs is not merely more transactional deployments of intelligence that may benefit only the transactors, but also, transformational deployments that will make the world into a better place.

A reviewer of this article suggested that scientific evidence for the “transformational-transactional” distinction is lacking. However, this distinction does not relate to kinds of intelligence—to factors, information-processing components, or loci in the brain that might be teased out, respectively, psychometrically, experimentally, or neuropsychologically. Rather, the distinction refers, as the title indicates, to *deployments* of intelligence—to how people use their intelligence. Do we need psychometric or other analyses to recognize that some people deploy their intelligence to garner rewards—grades, money, an ample house, a prestigious car—and that others use it to change the world? Do we need experiments to tell us that some people make money and give little or nothing back to the world—or worse, give negatively—and that other people change the world for the better? Do we need a biological analysis to tell us that there is a difference between the deployment of intelligence of people who use their high IQs to churn financial derivatives on Wall Street, and people who use their intelligence positively and meaningfully to change the world, like Mahatma Gandhi, Martin Luther King, Jr., and Abraham Lincoln? Are we so oblivious of our environmental contexts that we prefer to find “truth” in our artificially contrived studies rather than in the world around us? We do not know what the world-changers’ IQs may have been. We do know that they deployed their IQ and whatever else constitutes their intelligence to make the world a better place. I would suggest the reviewer may have it “backward.” We will never know through laboratory work who uses their intelligence transformationally: For that, we have to look to contributions to the world, not to responses on contrived laboratory tasks or educational tests.

James Flynn was transformationally gifted. He also in all likelihood had a sky-high IQ. However, it was not his IQ that made him transformationally gifted. Many people with sky-high IQs merely parrot in slightly different words or with slightly different empirical studies what so many others have said and done before them. For example, the field of giftedness has been locked into the century-old Terman paradigm, with few exceptions (e.g., Ambrose 2012; Gentry et al. n.d.; Renzulli 2012). What made Flynn transformationally gifted was his willingness to question orthodoxy (see Sternberg 2018)—both the assertions of his colleagues in the field of intelligence and the prevailing presuppositions (Zeitgeist) underlying those assertions, for example, that IQ is stable across secular time and that what matters is how much intelligence one has rather than how one deploys that intelligence. His transformational giftedness was in his mindset—in the way he used his intelligence. He opened his mind as could so many of us if we just only thought to do so.

## Figures and Tables

**Table 1:** STGS: Preliminary Version.

Part I	1. Write a paragraph about what your future dream life in 25 years would look like, with the constraint that there is a chance of achieving it. 2. What are you passionate about? How would you expect that passion to affect your future life? 3. Design an App. What is its purpose and how does it accomplish it?
Part II	1. What would you most like to accomplish in your life? How will you get from where you are to where you want to be to accomplish that thing? 2. What are two other things you would like to accomplish in your life? 3. When you are older, how will you decide if you are satisfied with what you have done in your life? 4. What do you see as the biggest obstacle to accomplishing your principal goal in life and how will you overcome it?
Part III	1. Pick a major world problem. What are things you personally could do to help solve the problem? How could you do them? 2. What are things the country in which you live could do to help solve the problem you chose? How could the country do them? 3. Have you done anything in your life that you believe helps to make the world a better place? If so, what?
Part IV	What is the one thing you have done in your life of which you are most proud? Why are you proud of it?
Part V	If you were to change one thing in the world, what would it be?
Part VI	1. Martin Luther King and Mahatma Gandhi both defied the laws of their times and went to prison for their beliefs. Did they do the right thing in defying the law? Why or why not? 2. Suppose you had a belief about how things in the world need to change. But other people close to you told you they disagreed with you. What would you do? 3. Do you think there might be a time in your life when it will be better to be right than to be well liked? If so, what might an example be?
Part VII	1. If a lot of people believe something, do you generally conclude that it is most likely true? Why or why not? 2. Have you had any beliefs that you used to accept but that you no longer accept? If so, what changed your mind, and why? 3. Can you think of a belief most people have that you do not accept? If so, what is it and why do you not accept it?

Scoring: Scoring is by the consensual assessment technique. Judges are asked to rate the extent to which each response reflects, on a 1–5 scale, a transformational rather than merely transactional mind-set.

## References

[B1-jintelligence-09-00015] Ambrose Donald, Ambrose D., Sternberg R. J., Sriraman B. (2012). The not-so-invisible hand of economics and its impact on conceptions and manifestations of high ability. Confronting Dogmatism in Gifted Education.

[B2-jintelligence-09-00015] Bass Bernard. M. (1998). Transformational Leadership: Industrial, Military, and Educational Impact.

[B3-jintelligence-09-00015] Bass Bernard M., Avolio Bruce. J. (1994). Improving Organizational Effectiveness through Transformational Leadership.

[B4-jintelligence-09-00015] Bradbury Thomas N., Karney Benjamin. R. (2019). Intimate Relationships.

[B5-jintelligence-09-00015] Burgat Casey, Hunt Charles (2018). Congress in 2019: The 2nd Most Educated and Least Politically Experienced House Freshman Class. https://www.brookings.edu/blog/fixgov/2018/12/28/congress-in-2019-the-2nd-most-educated-and-least-politically-experienced-house-freshman-class/.

[B6-jintelligence-09-00015] Carnes Nicholas, Lupu Noam (2015). What Good Is a College Degree? Education and Leader Quality Reconsidered. https://www.noamlupu.com/leader_education.pdf.

[B7-jintelligence-09-00015] Ceci Stephen J. (1996). On Intelligence.

[B8-jintelligence-09-00015] Dickens William T., Flynn James R. (2001). Heritability estimates versus large environmental effects: The IQ paradox resolved. Psychological Review.

[B9-jintelligence-09-00015] Flynn James R. (1984). The mean IQ of Americans: Massive gains 1932 to 1978. Psychological Bulletin.

[B10-jintelligence-09-00015] Flynn James R. (1987). Massive IQ gains in 14 nations. Psychological Bulletin.

[B11-jintelligence-09-00015] Flynn James R. (2007). What Is Intelligence? Beyond the Flynn Effect.

[B12-jintelligence-09-00015] Flynn James R. (2013). Intelligence and Human Progress: The Story of What Was Hidden in Our Genes.

[B13-jintelligence-09-00015] Gentry Marcia, Desmet Ophelie A., Karami Sareh, Lee Hyesong, Green Corinne, Cress Anne, Chowkase Aakash, Gray Andre (n.d.). Gifted Education’s Legacy of High Stakes Ability Testing: Using Measures for Identification that Perpetuate Inequity. Roeper Review.

[B14-jintelligence-09-00015] Gould Stephen. J. (1981). The Mismeasure of Man.

[B15-jintelligence-09-00015] Halberstram David (1993). The Best and the Brightest.

[B16-jintelligence-09-00015] Renzulli Joseph. S. (2012). Reexamining the role of gifted education and talent development for the 21st century: A four-part theoretical framework. Gifted Child Quarterly.

[B17-jintelligence-09-00015] Sandel Michael. J. (2020). The Tyranny of Merit: What’s Become of the Common Good? Farrar, Straus, and Giroux.

[B18-jintelligence-09-00015] Senior Jennifer (2020). 95 Percent of Representatives Have a Degree. Look Where That’s Got Us. New York Times.

[B19-jintelligence-09-00015] Sternberg Robert. J. (1997). Successful Intelligence.

[B20-jintelligence-09-00015] Sternberg Robert. J., Sternberg R. J., Jordan J. (2005). Foolishness. Handbook of Wisdom: Psychological Perspectives.

[B21-jintelligence-09-00015] Sternberg Robert. J. (2017). ACCEL: A new model for identifying the gifted. Roeper Review.

[B22-jintelligence-09-00015] Sternberg Robert. J. (2018). A triangular theory of creativity. Psychology of Aesthetics, Creativity, and the Arts.

[B23-jintelligence-09-00015] Sternberg Robert. J., Sisk D., Wallace B., Senior J. (2019a). Is gifted education on the right path? The ACCEL model of giftedness. Handbook of Gifted Education.

[B24-jintelligence-09-00015] Sternberg Robert. J. (2019b). A theory of adaptive intelligence and its relation to general intelligence. Journal of Intelligence.

[B25-jintelligence-09-00015] Sternberg Robert J., Cross T. L., Olszewski-Kubilius P. (2020a). Transformational giftedness. Conceptual Frameworks for Giftedness and Talent Development.

[B26-jintelligence-09-00015] Sternberg Robert J. (2020b). Transformational giftedness: Rethinking our paradigm for gifted education. Roeper Review.

[B27-jintelligence-09-00015] Sternberg Robert J. (2021). Adaptive Intelligence: Surviving and Thriving in a World of Uncertainty.

[B28-jintelligence-09-00015] Sternberg Robert J., Desmet Ophelie A., Ford Donna, Gentry Marcia L., Grantham Tarek, Karami Sareh (n.d.). The Legacy: Coming to Terms with the Origins and Development of the Gifted-Child Movement. Roeper Review.

[B29-jintelligence-09-00015] Sternberg Robert J., Sternberg R. J., Ambrose D., Karami S. (n.d.). Transformational giftedness: Who’s got it and who does not?. Transformational Giftedness.

[B30-jintelligence-09-00015] Terman Lewis. M. (1916). The Measurement of Intelligence: An Explanation of and a Complete Guide for the Use of the Stanford Revision and Extension of the Binet-Simon Intelligence Scale.

[B31-jintelligence-09-00015] Terman Lewis. M. (1925). Genetic Studies of Genius: Mental and Physical Traits of a Thousand Gifted Children.

[B32-jintelligence-09-00015] Tommasi Marco, Pezzuti Lina, Colom Roberto, Abad Francisco J., Saggino Aristede, Orsini Arturo (2015). Increased educational level is related with higher IQ scores but lower g-variance: Evidence from the standardization of the WAIS-R for Italy. Intelligence.

